# GRACy: A tool for analysing human cytomegalovirus sequence data

**DOI:** 10.1093/ve/veaa099

**Published:** 2020-12-30

**Authors:** Salvatore Camiolo, Nicolás M Suárez, Antonia Chalka, Cristina Venturini, Judith Breuer, Andrew J Davison

**Affiliations:** 1 MRC-University of Glasgow Centre for Virus Research, Glasgow, UK; 2 Division of Infection & Immunity, Roslin Institute, R(D)SVM, University of Edinburgh, Edinburgh, UK; 3 Division of Infection and Immunity, University College London, London, UK

**Keywords:** human cytomegalovirus, genotyping, de novo assembly, genome annotation, variant discovery

## Abstract

Modern DNA sequencing has instituted a new era in human cytomegalovirus (HCMV) genomics. A key development has been the ability to determine the genome sequences of HCMV strains directly from clinical material. This involves the application of complex and often non-standardized bioinformatics approaches to analysing data of variable quality in a process that requires substantial manual intervention. To relieve this bottleneck, we have developed GRACy (Genome Reconstruction and Annotation of Cytomegalovirus), an easy-to-use toolkit for analysing HCMV sequence data. GRACy automates and integrates modules for read filtering, genotyping, genome assembly, genome annotation, variant analysis, and data submission. These modules were tested extensively on simulated and experimental data and outperformed generic approaches. GRACy is written in Python and is embedded in a graphical user interface with all required dependencies installed by a single command. It runs on the Linux operating system and is designed to allow the future implementation of a cross-platform version. GRACy is distributed under a GPL 3.0 license and is freely available at https://bioinformatics.cvr.ac.uk/software/ with the manual and a test dataset.

## 1. Introduction

Human cytomegalovirus (HCMV; species *Human betaherpesvirus 5*) infects 60–70 per cent of adults in developed countries and up to 100 per cent in developing countries (Zuhair et al. 2019). Infection is often asymptomatic but can cause severe disease in immunodeficient or immunocompromised people such as neonates or transplant recipients (Griffiths et al. 2015). HCMV genomics has been significantly advanced in recent years by high-throughput sequencing, which has resulted in the publication of genome sequences for >250 strains. A significant number of these sequences have been derived directly from clinical material by target enrichment and Illumina sequencing (Houldcroft et al. 2016; Lassalle et al. 2016; Suárez et al. 2019a,b; Suárez et al. 2020). Processing the short-read data of variable quality obtained in this way into an annotated HCMV genome currently involves many steps with significant manual intervention and would benefit from a bioinformatics toolkit that automates and integrates the components.

The HCMV genome is a linear, double-stranded DNA molecule of approximately 236 kbp (Davison et al. 2013). Its overall configuration may be described as *ab*–U_L_–*b′a′c′*–U_S_–*ca*, in which U_L_ (193 kbp) and U_S_ (35 kbp) are unique regions flanked by inverted repeats *ab*/*b′a′* (1 kbp) and *a′c′*/*ca* (3 kbp); component sizes vary among strains. In addition, the 3′ end of each DNA strand terminates with an unpaired nucleotide: an A residue at the left end and a complementary T residue at the right end. To avoid assembly problems due to the inverted repeats, a genome is initially constructed from short-read data in a trimmed form that lacks most of *ab* at the left end and most of *ca* at the right end. The genome is completed by locating the sequences corresponding to the genome termini at the *b′a′* and *a′c′* junctions and adding the reverse complemented versions to the ends of the trimmed genome.

Challenges are also inherent in annotating an HCMV genome, which contains ≥170 tightly packed canonical protein-coding genes (Gatherer et al. 2011). In most strains, at least one of these genes is present as a pseudogene, commonly due to an in-frame termination codon or an insertion or deletion (indel) causing a frameshift (Sekulin et al. 2007; Cunningham et al. 2010; Sijmons et al. 2014, 2015; Suárez et al. 2019a). Also, although most genes are well conserved among strains, some are hypervariable, each existing as several distinct, stable genotypes (Pignatelli et al., 2004; Puchhammer-Stckl and Grzer 2011). Finally, recombination during HCMV evolution has essentially obliterated genetic linkage and generated a huge number of different strains (Rasmussen *et al*. 2003; Sijmons et al. 2015; Suárez et al. 2019a). These aspects of diversity limit the effectiveness of reference-guided genome assembly and of automatic transfer of annotations from a reference. 

Although hypervariable genes create challenges to annotation, they are useful for determining the number of HCMV strains present in a clinical sample. This is an important consideration, as multiple-strain infections exhibit far more diversity than single-strain infections, and failure to recognize their presence can compromise genome determination and result in gross overestimation of intrahost evolutionary rate (Hage et al. 2017; Cudini et al. 2019; Suárez et al. 2019a; Suárez et al. 2020). One approach to monitoring strain composition is to count the occurrences in the reads of a single sequence motif (21–24 nucleotides (nt)) that is specific to each genotype of a hypervariable gene and conserved in all known sequences of that genotype (Suárez et al. 2019a,b). Although this approach is largely effective, its blindness to occasional new polymorphisms in the motifs indicates a need for a more sensitive approach. Monitoring strain composition also provides a means of detecting apparent cross-contamination of samples, which may occur at any of the steps involved in sample processing, library preparation, and sequencing. In addition, the bioinformatic steps involved in assembling and analysing HCMV sequence data must take into account the vicissitudes of data quality.

Here, we present GRACy (Genome Reconstruction and Annotation of Cytomegalovirus), a toolkit for analysing HCMV sequence data that addresses the shortcomings of current approaches. GRACy is written in Python and is embedded in a graphical user interface (GUI) with all required dependencies installed by a single command. It runs on the Linux operating system and is designed to allow the future implementation of a cross-platform version. The GUI presents six modules: read filtering, genotyping, genome assembly, genome annotation, variant analysis, and database submission. These modules were tested extensively on simulated and experimental data and outperformed generic approaches. We intend GRACy to provide an easy-to-use, expandable toolkit in support of HCMV genomics research.

## 2. Materials and methods

### 2.1 Datasets

Four primary datasets were produced *in silico* to mimic sequence data for single HCMV strains generated using the Illumina platform (Supplementary Table S1). Two of these simulated even coverage (EC) depth of the HCMV genome, and two simulated uneven coverage (UC) depth in order to resemble typical experimental data, which are highly influenced by local effects on the efficiency of target enrichment and polymerase chain reaction (PCR) amplification. Datasets merlin_EC_ and merlin_UC_ were generated from the strain Merlin reference sequence (Merlin; GenBank accession AY446894.2) using ART 2.5.8 (Huang et al. 2012). Each consisted of 150 nt paired-end reads derived from genome fragments having an average size of 500 nt (SD = 50 nt) and a sequence error profile typical of the Illumina HiSeq 2500 platform. Dataset merlin_UC_ was derived by dividing Merlin into 1,000 nt fragments using an adjacent window approach (window step = 500 nt). Paired-end reads were then generated from each window with a mean coverage depth chosen randomly between 5 and 4,000 reads/nt, and pooled. Datasets merlinVar_EC_ and merlinVar_UC_ were produced similarly and mimicked data for a different HCMV strain, the reference sequence of which (MerlinVar) was produced by using SimuG 1.0 (Yue et al. 2019) to introduce 4000 random substitutions into Merlin; this number is typical of the difference between two HCMV strains (Suárez et al. 2019a). Four series of subsampled datasets (e.g. ${\text{merlin}}_{\text{EC}}^{20k}$ containing 20,000 paired-end reads from merlin_EC_) were then derived randomly from the primary datasets (Supplementary Table S2). Also, datasets simulating mixtures of the two HCMV strains were generated by concatenating ${\text{merlin}}_{\text{EC}}^{3200k}$ with various proportions of reads from merlinVar_EC_ or by concatenating ${\text{merlin}}_{\text{UC}}^{3200k}$ with various proportions of reads from merlinVar_UC_ (e.g. ${\text{mixedStrains}}_{\text{EC}}^{28k}$ contained 28,000 paired-end reads from merlinVar_EC_, which thus comprised 0.9% of the total; Supplementary Table S3).

In addition to the simulated datasets, seven experimental datasets derived directly from clinical material by target enrichment and Illumina sequencing were downloaded from public databases (Supplementary Table S4). Four originated from congenitally infected infants whose amniotic fluid (JER4755, PAV6, and PAV25) or urine (JER851) had been sampled (Suárez et al. 2019a). Three (LON2_T1, LON2_T2, and LON2_T3) originated from blood samples from an HCMV-positive infant collected at different time points during infection (Houldcroft et al. 2016). All samples had been characterized as containing a single strain, and genotyping data, genomes and annotations were available (Houldcroft et al. 2016; Suárez et al. 2019a).

### 2.2 Performance statistics

Where appropriate, modules were evaluated in terms of false discovery rate (FDR) and sensitivity. FDR was calculated as the ratio between the number of false-positive values (FP) and the sum of FP and true-positive values (TP), and sensitivity was calculated as the ratio between TP and TP plus false-negative values (FN).

For the genome assembly module, the sequence produced by GRACy was aligned with the expected genome (Merlin or MerlinVar for the simulated data, and the deposited genome for the experimental data) using MAFFT v. 7.310 (Katoh 2002). In this context, TP corresponded to nucleotides that matched in the alignment, FP to nucleotides that were present only in the sequence produced by GRACy, and FN to nucleotides that were present only in the expected sequence. Sequences produced by GRACy were also evaluated by computing N50 values, which are a measure of assembly continuity (Earl et al. 2011), and by counting the number of undetermined nucleotides (N). For the experimental data, GRACy was compared with the viral genome *de novo* assembly pipeline vrap (https://www.rna.uni-jena.de/research/software/vrap-viral-assembly-pipeline/).

For the variant analysis module, TP, FP, and FN were considered to be the number of single nucleotide polymorphisms (SNPs) that were simulated and detected correctly, not simulated and detected incorrectly, and simulated and not detected, respectively.

For the annotation module, the numbers of exons and their predicted coordinates were compared withthose in published data.

## 3. Software implementation

The main steps of each GRACy module are described below **(**further details are provided in Supplementary File S1). Each module is embedded within a GUI written using the Qt graphics library and can be run independently. Output data are generated in the form of text files or plots (Supplementary Table S5). Third-party software components were used with default parameters unless otherwise specified.

### 3.1 Read filtering

Despite the use of target enrichment, a dataset from a sample with low HCMV load may contain a significant proportion of human reads. The viral reads may also be highly clonal owing to excessive PCR amplification, and this may compromise the genome and, particularly, subsequent variant analysis. In addition, the reads may contain portions of the adapters incorporated during library preparation, especially when the fragments are shorter than the anticipated read length. In this module, an original dataset undergoes the following steps: 1) removal of human reads using Bowtie 2 v. 2.3 (Langmead and Salzberg 2012; Keel and Snelling 2018) under the *–local* and –*end-to-end* options with the human reference genome (Hg38; http://bowtie-bio.sourceforge.net/bowtie2/manual.shtml); 2) trimming of default or user-specified adapters and low-quality nucleotides using Trim Galore v. 0.6.4 (bioinformatics.babraham.ac.uk); 3) removal (deduplication) of clonal reads using FastUniq v. 1.1 (Xu et al. 2012), which detects duplicates on the basis of read pairs sharing the same sequences; and 4) alignment of reads to Merlin using Bowtie 2 to derive data on coverage breadth (the proportion of the genome covered by reads) and mean coverage depth (reads/nt) of positions matching ≥1 read.

Alternative methods for read filtering are used by the genome assembly and variant analysis modules.

### 3.2 Genotyping

**Figure 1.**
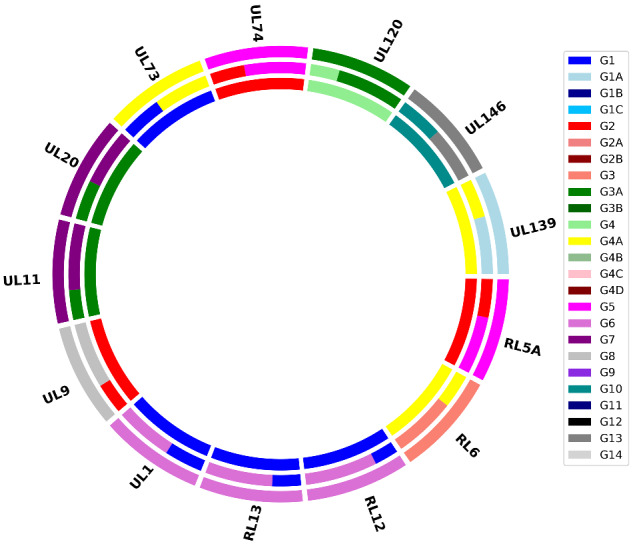
Doughnut plot reporting the results of a genotyping analysis. Each dataset is depicted as an annulus divided into sections representing the 13 hypervariable genes analysed. The size of the coloured bars within each section indicate the proportion of the genotypes detected, according to the key on the right. The analyses of three datasets are plotted concentrically to facilitate comparison.

This module uses a motif-based approach to genotype 13 hypervariable HCMV genes (in order along the genome: RL5A, RL6, RL12, RL13, UL1, UL9, UL11, UL20, UL73, UL74, UL120, UL146, and UL139). In contrast to using a single motif for each genotype (Suárez et al. 2019a,b), it utilizes all possible motifs (as kmers, k = 17) for each genotype based on publicly available HCMV genomes, and therefore the number of kmers for a genotype is variable, ranging from 2 (UL73/G4C) to 1007 (RL12/G5) (Supplementary Fig. S1). All kmers in the reads are counted, and a dictionary is created using Jellyfish v. 2.2 (Marçais and Kingsford 2011), which associates each kmer with the reads in which it is present. The dictionary is searched using Jellyfish for genotype-specific kmers derived from a large collection of HCMV genomes, and the relevant reads are listed non-redundantly and enumerated. If the number of reads for a genotype exceeds a user-specified proportion of the average coverage depth of the genome, the data are reported as the ratio between the number of reads identifying each individual genotype divided by the total number of reads identifying any genotype for that gene. These values are reported in a text file and presented visually as doughnut plots, and may be used for strain enumeration (Suárez et al. 2019a) (Fig. 1).

### 3.3 Genome assembly

This module constructs the genome from the original datasets. The paired-end dataset files are quality filtered using PRINSEQ v. 0.20 (Schmieder and Edwards 2011) and interleaved, and the resulting single dataset is normalized using scripts from the khmer v. 3.0 suite (Crusoe et al. 2015) to reduce the negative impact of uneven genome coverage depth on subsequent steps (Brown et al. 2012). Since dataset subsampling is known to improve the efficiency of *de novo* assembly (Hug 2018), several subsampled datasets containing 20–100 per cent of the reads selected randomly are assembled individually into contigs using SPAdes v. 3.12 (Bankevich et al. 2012). The contig set with the highest N50 value is aligned to Merlin in order to determine contig position and orientation, and the contigs are joined using a combination of Scaffold_builder v. 2.0 (Silva et al. 2013) and Ragout v. 2.2 (Kolmogorov et al. 2014) to form a scaffold with gaps corresponding to the regions that failed *de novo* assembly. These gaps are resolved by locating the flanking 100 nt regions in a large collection of HCMV genomes using blastn v. 2.9. If close similarity is found to the same genome for both flanking regions, the reads are aligned to a sequence consisting of these regions and the intervening sequence, differences indicated by the alignment are corrected, and the consensus is incorporated into the genome. Attempts are made to fill any remaining gaps using GapFiller 1.11 (Boetzer and Pirovano 2012).

At this stage, the internal repeat region containing the *b′a′* and *a′c′* sequences (especially *b′*) frequently contains errors, and an attempt is made to improve the sequence. The reads are aligned to the internal repeat region in Merlin (approximately 10,000 nt), the read pairs for which at least one member is aligned are extracted and assembled *de novo* using SPAdes, and the contigs are scaffolded against the internal repeat region in Merlin using Scaffold_builder. This step generally results in a more reliable version of the internal repeat region that is used to replace the original one.

The reads are then aligned to the genome using Bowtie 2, and the coverage depth is calculated at each position. Regions resulting from potential misassembly errors are detected as having low or zero coverage depth and are removed from the assembly, thus creating gaps, which are filled using an internal algorithm that exploits read overlap to extend the flanking regions, a method known as overlap/layout/consensus (OLC). The terminal *ab* and *ca* inverted repeats are generated by reverse complementing the internal *b′a′* and *a′c′* sequences. If either of these sequences does not overlap the genome (which is in trimmed form at this stage), gaps are introduced and filled using OLC. Finally, major variants (substitutions or indels, the latter including variations in homopolymer length) are detected using Varscan v. 2.4 (Koboldt et al. 2012) and, if necessary, the genome is amended to contain the most frequent variants. This step also corrects any residual errors in the genome.

### 3.4 Genome annotation

This module predicts the functional protein-coding sequences (CDSs) in a genome, based on those annotated in Merlin. Briefly, the amino acid sequences of the 170 canonical proteins are mapped to the genome using tblastn v. 2.9 (Altschul et al. 1990). The best-matching variant for each protein in Uniprot (https://www.uniprot.org; accessed 1 December 2019) is then identified and mapped using exonerate v. 2.4 (Slater and Birney 2005), which is able to annotate splice sites and potential disruptions. This two-step approach is much faster than using exonerate alone. After retrieving the CDS coordinates, an internal algorithm performs a quality control on the annotation. If a CDS does not start with an ATG start codon or end with a stop codon (TAA, TGA, or TAG), due, for example, to substitutions in these triplets, the annotation is refined by searching for a nearby start or stop codon in the appropriate reading frame. If a valid annotation is not found at this stage, the exonerate alignment is repeated under the *-refine full* option (a more time-consuming dynamic programming strategy), followed, if necessary, by a further round of refinement to identify start and stop codons. The output files are fasta files for the CDS nucleotide and amino acid sequences, a genome annotation gff3 file, and a log file reporting disruptive mutations and refinements of start and stop codons.

### 3.5 Variant analysis

This module takes a conservative approach to automating the alignment of reads to a genome in order to detect variants. The original reads are trimmed using Trim Galore, quality-filtered using PRINSEQ under stringent parameters (*-min_qual_mean 25 -trim_qual_right 30 -trim_qual_window 5 -trim_qual_step 1 -min_len 80 -trim_ns_right 20*), and aligned to the genome using Bowtie 2. The aligned reads are extracted and deduplicated using Picard v. 2.21 (http://broadinstitute.github.io/picard), which marks paired-end reads sharing the same coordinates in the alignment. Nucleotides with a Phred quality score of ≥30 form the basis for calling SNPs using LoFreq v. 2.1.4 (Wilm et al. 2012), which considers one read pair for each marked duplicate. The module reports the position and frequency of each SNP, the name of the affected gene, the altered codon and, for a non-synonymous SNP, the amino acid substitution. The results can be filtered easily to focus on mutations in specific genes (e.g. those involved in antiviral drug resistance) or disruptive mutations, or to divide SNPs into synonymous and nonsynonymous categories.

### 3.6 Database submission

This module automates the submission of read datasets to the European Nucleotide Archive (ENA) short-read database. The user provides basic information in tabular format on the samples, the sequencing protocol, and the project under which the samples are submitted. The module produces intermediate XML files and submits the datasets using Webin-cli v. 3.0. The submission can be directed to the ENA test web space, thus allowing checks to be carried out before submission to the official ENA database.

## 4. Software evaluation using simulated data

### 4.1 Genotyping

The performance of the multi-motif approach in this module was compared with the single-motif approach using three of the datasets subsampled from merlin_UC_ and merlinVar_UC_ (Supplementary Table S2). As anticipated, the multi-motif approach reported more matching reads for each genotype (Supplementary Table S6). Both approaches were successful with all merlin_UC_ datasets, but the multi-motif approach was more effective for the merlinVar_UC_ datasets, failing only for genotype UL73, probably due to the low number of motifs specific for genotype G4D (Supplementary Fig. S1), whereas the single-motif approach failed in genotyping six genes. Detection of artefactual kmers was more prominent with larger datasets, especially for the multi-motif approach, probably as a result of simulated sequencing errors. For this reason, this module implements a user-defined cut-off value for filtering out genotypes detected in extremely low proportions.

### 4.2 Genome assembly

This module was tested using the datasets subsampled from merlin_UC_ and merlinVar_UC_ (Supplementary Table S2). It produced a single scaffold covering the complete Merlin genome from merlin_UC_-derived datasets containing ≥40,000 reads but not from one containing 20,000 reads (Supplementary Table S7). The lower efficiency for the latter dataset was due to portions of the merlin genome that were not represented in ${\text{merlin}}_{\text{UC}}^{20k}$ because of UC (Supplementary Table S2).

Assembly of datasets from samples containing multiple HCMV strains is more challenging due to the presence of reads derived from variants having highly similar sequences. The ability of the module to assemble Merlin was tested using the datasets simulating two mixed strains containing merlin_EC_-derived reads and a proportion of merlinVar_EC_-derived reads, or merlin_UC_-derived reads and a proportion of merlinVar_UC_-derived reads, with the proportion ranging from 0.9 to 25.9 per cent (Supplementary Table S3). A scaffold representing the complete Merlin genome was produced for all datasets with high sensitivity and low FDR (Supplementary Table S8). The lower sensitivity observed for the highest proportion (25.9%) of merlinVar_EC_-derived reads, in comparison with merlinVar_UC_-derived reads, may have been due to the effects of UC depth.

Genome assembly was typically completed in <90 minutes on an Intel(R) Xeon(R) E7-4890 CPU running at 2.80 GHz with a maximum of 16 threads. The longest times were observed for lower coverage datasets due to the increased number of gap-closing attempts, and at higher coverage depth due to the more intensive computation required for higher numbers of reads.

### 4.3 Variant calling

This module was also evaluated using the datasets simulating two mixed strains (Supplementary Table S3). An FDR of 0 was achieved for all datasets analysed, whereas sensitivity depended on the proportion of the minor strain (Supplementary Table S3). The module reported >97 per cent of simulated SNPs for the merlin_EC_/merlinVar_EC_ series when the proportion of the second strain was 1.7 per cent, and >99.9 per cent when it was higher (Supplementary Fig. S2). A similar trend was observed for the merlin_UC_/merlinVar_UC_ series, but sensitivity never exceeded 90 per cent, which may again have been due to the effects of UC depth.

### 4.4 Annotation

This module was evaluated using Merlin, and produced CDS coordinates in accordance with those in the reference annotation. In this annotation, gene UL30A is unusual in featuring an alternative start codon (ATA), and therefore was recorded by the module as a pseudogene lacking a start codon (ATG). Annotation of merlinVar retrieved all the relevant CDSs, with a report on the amino acid sequence differences from Merlin and the presence of several pseudogenes due to in-frame stop codons or nonsynonymous mutations in start or stop codons.

## 5. Software evaluation using experimental data

### 5.1 Genotyping

As with simulated data, the multi-motif approach implemented in this module proved superior to the single-motif approach. For example, the former approach classified gene UL1 as genotype G3 in JER851 on the basis of 2098 reads, but the latter did not identify reads matching this or any other genotype (Supplementary Table S9), due to deletion of a 218 nt region containing the genotype G3 single motif. The module also improved the classification of low coverage datasets such as Lon2_T1. For gene UL9, the single-motif approach identified genotype G9 on the basis of 2 reads (Suárez et al. 2019a), whereas genotype G1 was identified in datasets from the other two samples from the same patient (Lon2_T2 and Lon2_T3). The module produced a more consistent and well-supported classification, with genotype G1 being identified in all Lon2 datasets (Supplementary Table S9).

### 5.2 Genome assembly

**Figure 2.**
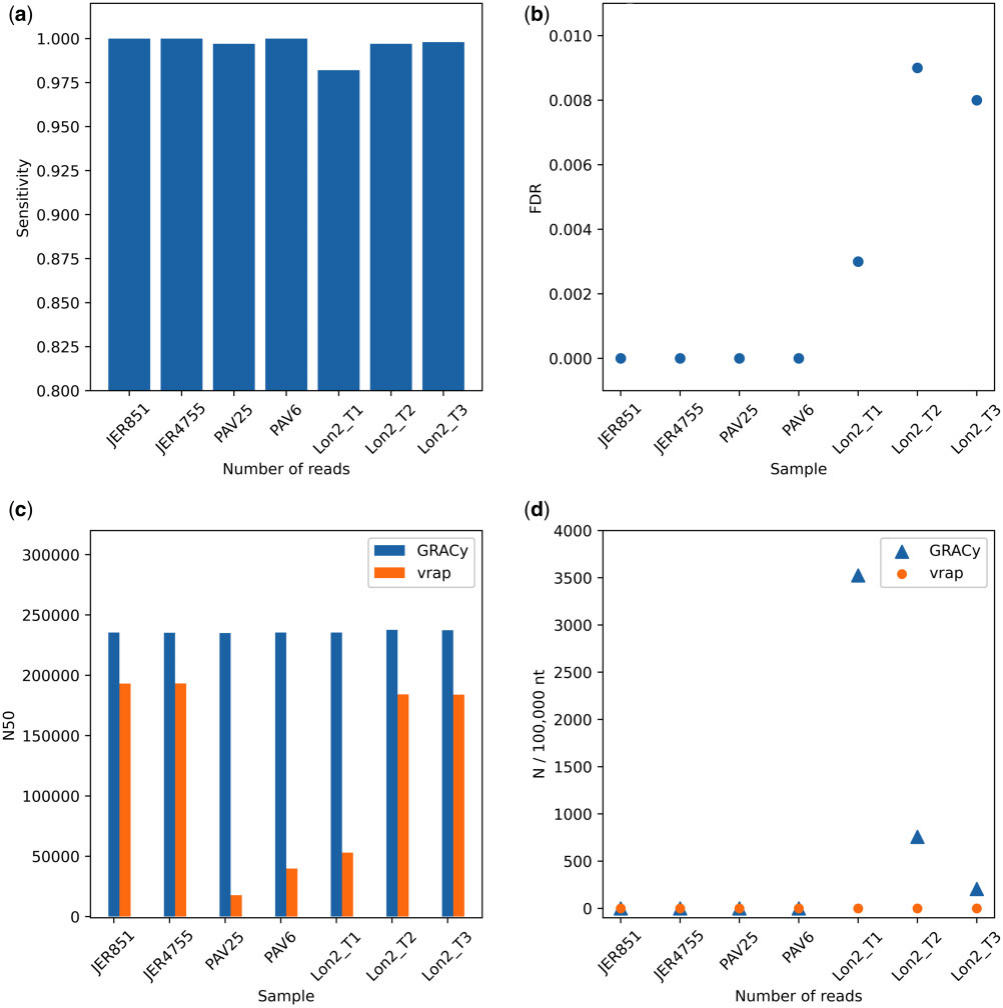
Genome assembly statistics for experimental datasets: (a) sensitivity, (b) FDR, (c) N50 values and (d) unidentified nucleotides (N).

This module was compared with the virus genome assembly pipeline vrap, and found to be superior. The module produced a single scaffold for all datasets, with a sensitivity ranging between 98.20 per cent (Lon2_T1) and 100 per cent (JER851, JER4755, and PAV6) (Fig. 2A). FDR ranged from 0 (JER4755, JER851, PAV25, and PAV6) to 0.009 (Lon2_T2) (Fig. 2B), with higher values due to misassembly in the inverted repeat regions (Supplementary Table S10). The module was also superior in terms of N50 value, improving this by between 42,014 nt (JER4755) and 217,313 nt (PAV25) (Fig. 2C). However, it was not generally superior in terms of number of unidentified nucleotides in the genome (Fig. 2D). Overall, execution times were in line with those observed for simulated datasets.

Each difference between the genome generated by this module and the corresponding deposited genome was investigated by counting the number of reads supporting the alternatives in datasets deduplicated using Picard (Supplementary Table S10). Indels were the most common difference and always occurred in homopolymeric tracts, which are prone to length variation during viral replication and because of DNA polymerase slippage during PCR. The sequences produced by GRACy were supported by a higher number of reads in 14/16 instances, the two exceptions in Lon2_T1 being due to insufficient coverage depth preventing improvement during the last step of genome assembly. The *ab* inverted repeats in PAV25 were misassembled by GRACy, resulting in the absence of the first 698 nt of the genome, and an extraneous 1939 nt sequence was added at the left end of the genome in Lon2_T2 and Lon2_T3. Several SNPs and indels were reported in a region of the PAV25 genome (positions 140703–140728) consisting of tandem GGT repeats in a G-rich context (Supplementary Fig. S3), which is a situation known to promote sequencing errors (Meacham et al. 2011). Finally, the nucleotide alternative to that in the deposited genome was called by the module for nine positions with SNPs in Lon_T1, and each was supported by the highest number of reads.

### 5.3 Variant calling

**Figure 3.**
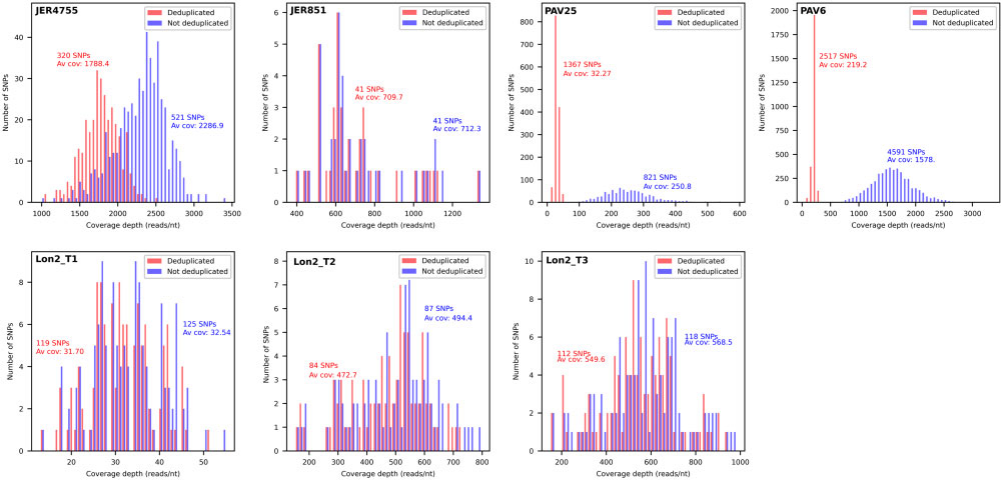
Distribution of the numbers of SNPs in experimental datasets as a function of coverage depth (reads/nt). The distributions are shown for the original (blue) and deduplicated (red) datasets. Av cov, average coverage.

This module was used to investigate the genetic variability represented in the datasets. The reads were aligned to the deposited genome with or without deduplication using Picard, and variants were called (Fig. 3). In general, a lower number of SNPs was detected for deduplicated reads, as PCR-derived duplicates tend to lead to an increase in the number of reads supporting variant calls (DePristo et al. 2011). However, an unusually high number of SNPs was called for deduplicated PAV25, presumably because deduplication led to a significant decrease in average coverage depth (from >250 to <50 reads/nt), a scenario in which contaminating reads from other libraries with less clonality sequenced on the same run tend to become detectable. The number of variants was linked to the number of strains represented in the datasets, as reads originating from a minor strain result in many SNPs when aligned to the genome of the major strain. Indeed, PAV25 and PAV6 were predicted to contain reads from additional strains, probably originating from contamination, with the deeper sequencing of the latter enhancing the number of SNPs.

The output files from the module also provided insights into the possible biological relevance of some SNPs. For example, analysis of Lon2_T3 revealed 1 and 7 nonsynonymous mutations in genes UL97 and UL54, respectively, with which resistance to anti-HCMV drugs is associated (Table 1). Indeed, patient Lon2 was reported to have developed resistance mutations in these two genes during antiviral treatment (Houldcroft et al. 2016). The module detected these mutations plus two others in UL54 that resulted in the amino acid changes E756D and R904L, the former having been reported previously as a resistance mutation (Lurain and Chou 2010). The greater sensitivity of the module to SNPs may be due to operation of the read filtering module prior to variant calling.

**Table 1.** SNPs detected by the variant-calling module in genes UL97 and UL54 for dataset Lon2_T3.

Gene	Position in genome	Position in protein	Frequency (%)	Nucleotide change	Codon change	Amino acid change
*UL97*	*142*,*842*	460	*11.5*	*T → G*	*ATT → ATG*	*I → M*
*UL54*	*78*,*631*	987	*1.6*	*C → T*	*GCC → GTC*	*A → V*
UL54	78,877	905	2.8	G *→* T	CGG *→* CTG	R *→* L
*UL54*	*79*,*165*	809	*5.0*	*C → T*	*GCG → GTG*	*A → V*
*UL54*	*79*,*187*	802	*4.3*	*C → A*	*CTG → ATG*	*L → M*
UL54	79,323	756	2.3	G *→* T	GAG *→* GAT	E *→* D
*UL54*	*79*,*858*	578	*14.8*	*T → A*	*CTG → CAG*	*L → Q*
*UL54*	*80*,*052*	513	*4.2*	*T → G*	*AAT → AAG*	*N → K*

Variants previously reported in this dataset (Houldcroft et al. 2016b) are shown in italic font.

### 5.4 Genome annotation

This module was tested on the deposited genomes (Supplementary Table S4). The resulting annotations differed minimally from the originals (Supplementary Table S11). Gene UL30A was always reported as a pseudogene due to its non-canonical start codon (ATA), and differences in the annotations of genes RL5A and RL6 were observed when a start or a stop codon could not be identified. Additional differences were observed when alternative start codons were present close together at the start of a CDS. For example, slightly longer CDSs were reported for gene UL6 in JER4755 and gene UL72 in PAV25. Minor variations of this sort are also apparent in deposited annotations.

## 6. Conclusion

GRACy was developed with the benefit of having analysed HCMV sequence data by more laborious methods and takes into account the characteristics of the HCMV genome and the limitations of sequence data obtained by target enrichment and Illumina sequencing. The six modules within the toolkit were tested and verified extensively on simulated and experimental datasets. The genotyping module employs a multi-motif approach that is superior to the single-motif approach used in previous studies. The genome assembly module features several steps aimed at exploiting the data within the bounds of their quality, and showed better performance in comparison with earlier routines. The genome annotation and data submission modules automate a process that, due to the degree of variation among HCMV strains, had previously required extensive manual intervention. The variant analysis module provides a means of detecting minority genomes with maximum stringency and specificity, and produces output files suitable for biological interpretation.

GRACy has been designed with future capabilities in mind. Underlying improvements will arise from the availability of additional genomes, including better definitions of genotype-specific kmers, the use of additional HCMV genes for genotyping and greater likelihood of closing gaps. They will also address features of the HCMV genome that present challenges to automated analysis, such as resolution of regions that are difficult to sequence (e.g. the inverted repeat regions), the inclusion of alternative start codons (gene UL30A), the resolution of how certain genes (RL5A and RL6) are translated and the incorporation of additional annotations of the Merlin reference. The package will also incorporate further tools for analysing HCMV sequence data. Finally, it is possible that the approach taken with GRACy will also be applicable to intensive genomic studies on other large DNA viruses. 

## Data availability

GRACy is distributed under a GPL 3.0 license and is freely available at https://bioinformatics.cvr.ac.uk/software/.

## Supplementary data

Supplementary data are available at *Virus Evolution* online.

## Supplementary Material

veaa099_Supplementary_Data

## References

[veaa099-B1] Altschul S. F. et al (1990) ‘ Basic Local Alignment Search Tool’, Journal of Molecular Biology, 215: 403–10. 2231712 10.1016/S0022-2836(05)80360-2

[veaa099-B2] Bankevich A. et al (2012) ‘ SPAdes: A New Genome Assembly Algorithm and Its Applications to Single-Cell Sequencing’, Journal of Computational Biology, 19: 455–77. 22506599 10.1089/cmb.2012.0021 PMC3342519

[veaa099-B3] Boetzer M., Pirovano W. (2012) ‘ Toward Almost Closed Genomes with GapFiller’, Genome Biology, 13: R56. 22731987 10.1186/gb-2012-13-6-r56 PMC3446322

[veaa099-B4] Brown C. T. et al (2012) ‘A Reference-Free Algorithm for Computational Normalization of Shotgun Sequencing Data’, arXiv:1203.4802.

[veaa099-B5] Crusoe M. R. et al (2015) ‘ The Khmer Software Package: Enabling Efficient Nucleotide Sequence Analysis’, F1000Research, 4: 900. 26535114 10.12688/f1000research.6924.1 PMC4608353

[veaa099-B6] Cudini J. et al (2019) ‘ Human Cytomegalovirus Haplotype Reconstruction Reveals High Diversity Due to Superinfection and Evidence of within-Host Recombination’, Proceedings of the National Academy of Sciences, 116: 5693–8. 10.1073/pnas.1818130116 PMC6431178 30819890

[veaa099-B7] Cunningham C. et al (2010) ‘ Sequences of Complete Human Cytomegalovirus Genomes from Infected Cell Cultures and Clinical Specimens’, Journal of General Virology, 91: 605–15. 10.1099/vir.0.015891-0 PMC2885759 19906940

[veaa099-B8] Davison A. J. et al (2013) ‘Comparative Genomics of Primate Cytomegaloviruses’, in Reddehase, M. J. (ed.) Cytomegaloviruses: From Molecular Pathogenesis to Intervention, pp. 1–22. London: Caister Academic Press.

[veaa099-B9] DePristo M. et al (2011) ‘ A Framework for Variation Discovery and Genotyping Using Next-Generation DNA Sequencing Data’, Nature Genetics, 43: 491–8. 21478889 10.1038/ng.806 PMC3083463

[veaa099-B10] Earl D. et al (2011) ‘ Assemblathon 1: A Competitive Assessment of de Novo Short Read Assembly Methods’, Genome Research, 21: 2224–41. 21926179 10.1101/gr.126599.111 PMC3227110

[veaa099-B11] Gatherer D. et al (2011) ‘ High-Resolution Human Cytomegalovirus Transcriptome’, Proceedings of the National Academy of Sciences of Sciences, 108: 19755–60. 10.1073/pnas.1115861108 PMC3241806 22109557

[veaa099-B12] Griffiths P., Baraniak I., Reeves M. (2015) ‘ The Pathogenesis of Human Cytomegalovirus’, The Journal of Pathology, 235: 288–97. 25205255 10.1002/path.4437

[veaa099-B13] Hage E. et al (2017) ‘ Characterization of Human Cytomegalovirus Genome Diversity in Immunocompromised Hosts by Whole-Genome Sequencing Directly from Clinical Specimens’, The Journal of Infectious Diseases, 215: 1673–83. 28368496 10.1093/infdis/jix157

[veaa099-B14] Houldcroft C. J. et al (2016) ‘ Detection of Low Frequency Multi-Drug Resistance and Novel Putative Maribavir Resistance in Immunocompromised Pediatric Patients with Cytomegalovirus’, Frontiers in Microbiology, 7: 1317. 27667983 10.3389/fmicb.2016.01317 PMC5016526

[veaa099-B15] Huang W. et al (2012) ‘ ART: A Next-Generation Sequencing Read Simulator’, Bioinformatics, 28: 593–4. 22199392 10.1093/bioinformatics/btr708 PMC3278762

[veaa099-B16] Hug L. A. (2018) ‘Subsampled Assemblies and Hybrid Nucleotide Composition/Differential Coverage Binning for Genome-Resolved Metagenomics’, in Walker, J. M. (ed.) Methods in Molecular Biology, Vol. 1849, pp. 215–25. New York: Humana Press Inc. 30298257 10.1007/978-1-4939-8728-3_14

[veaa099-B17] Katoh K. (2002) ‘ MAFFT: A Novel Method for Rapid Multiple Sequence Alignment Based on Fast Fourier Transform’, Nucleic Acids Research, 30: 3059–66. 12136088 10.1093/nar/gkf436 PMC135756

[veaa099-B18] Keel B. N., Snelling W. M. (2018) ‘ Comparison of Burrows-Wheeler Transform-Based Mapping Algorithms Used in High-Throughput Whole-Genome Sequencing: Application to Illumina Data for Livestock Genomes’, Frontiers in Genetics, 9: 35. 29535759 10.3389/fgene.2018.00035 PMC5834436

[veaa099-B19] Koboldt D. C. et al (2012) ‘ VarScan 2: Somatic Mutation and Copy Number Alteration Discovery in Cancer by Exome Sequencing’, Genome Research, 22: 568–76. 22300766 10.1101/gr.129684.111 PMC3290792

[veaa099-B20] Kolmogorov M. et al (2014) ‘ Ragout - A Reference-Assisted Assembly Tool for Bacterial Genomes’, Bioinformatics, 30: i302–9. 24931998 10.1093/bioinformatics/btu280 PMC4058940

[veaa099-B21] Langmead B., Salzberg S. L. (2012) ‘ Fast Gapped-Read Alignment with Bowtie 2’, Nature Methods, 9: 357–9. 22388286 10.1038/nmeth.1923 PMC3322381

[veaa099-B22] Lassalle F. et al (2016) ‘ Islands of Linkage in an Ocean of Pervasive Recombination Reveals Two-Speed Evolution of Human Cytomegalovirus Genomes’, Virus Evolution, 2: vew017. 30288299 10.1093/ve/vew017 PMC6167919

[veaa099-B23] Lurain N. S., Chou S. (2010) ‘ Antiviral Drug Resistance of Human Cytomegalovirus’, Clinical Microbiology Reviews, 23: 689–712. 20930070 10.1128/CMR.00009-10 PMC2952978

[veaa099-B24] Marçais G., Kingsford C. (2011) ‘ A Fast, Lock-Free Approach for Efficient Parallel Counting of Occurrences of k-Mers’, Bioinformatics, 27: 764–70. 21217122 10.1093/bioinformatics/btr011 PMC3051319

[veaa099-B25] Meacham F. et al (2011) ‘ Identification and Correction of Systematic Error in High-Throughput Sequence Data’, BMC Bioinformatics, 12: 451. 22099972 10.1186/1471-2105-12-451 PMC3295828

[veaa099-B26] Milne I. et al (2013) ‘ Using Tablet for Visual Exploration of Second-Generation Sequencing Data’, Briefings in Bioinformatics, 14: 193–202. 22445902 10.1093/bib/bbs012

[veaa099-B27] Pignatelli S. et al (2004) ‘ Genetic Polymorphisms among Human Cytomegalovirus (HCMV) Wild-Type Strains’, Reviews in Medical Virology, 14: 383–410. 15386592 10.1002/rmv.438

[veaa099-B28] Puchhammer-Stckl E., Grzer I. (2011) ‘ Human Cytomegalovirus: An Enormous Variety of Strains and Their Possible Clinical Significance in the Human Host’, Future Virology, 6: 259–71.

[veaa099-B2547864] Rasmussen L. Geissler A. Winters M. (2003) ‘ Inter‐ and Intragenic Variations Complicate the Molecular Epidemiology of Human Cytomegalovirus’, *The Journal of Infectious Diseases*, 187: 809–19. 12599055 10.1086/367900

[veaa099-B29] Schmieder R., Edwards R. (2011) ‘ Quality Control and Preprocessing of Metagenomic Datasets’, Bioinformatics, 27: 863–4. 21278185 10.1093/bioinformatics/btr026 PMC3051327

[veaa099-B30] Sekulin K. et al (2007) ‘ Analysis of the Variability of CMV Strains in the RL11D Domain of the RL11 Multigene Family’, Virus Genes, 35: 577–83. 17823862 10.1007/s11262-007-0158-0

[veaa099-B31] Sijmons S. et al (2014) ‘ A Method Enabling High-Throughput Sequencing of Human Cytomegalovirus Complete Genomes from Clinical Isolates’, PLoS One, 9: e95501. 24755734 10.1371/journal.pone.0095501 PMC3995935

[veaa099-B32] Sijmons S. et al (2015) ‘ High-Throughput Analysis of Human Cytomegalovirus Genome Diversity Highlights the Widespread Occurrence of Gene-Disrupting Mutations and Pervasive Recombination’, Journal of Virology, 89: 7673–95. 25972543 10.1128/JVI.00578-15 PMC4505652

[veaa099-B33] Silva G. G. Z. et al (2013) ‘Combining de Novo and Reference-Guided Assembly with Scaffold_Builder’, Source Code for Biology and Medicine, 8:. 23. 24267787 10.1186/1751-0473-8-23 PMC4177539

[veaa099-B34] Slater G. S. C., Birney E. (2005) ‘ Automated Generation of Heuristics for Biological Sequence Comparison’, BMC Bioinformatics, 6: 31. 15713233 10.1186/1471-2105-6-31 PMC553969

[veaa099-B35] Suárez N. M. et al (2019a) ‘ Human Cytomegalovirus Genomes Sequenced Directly from Clinical Material: Variation, Multiple-Strain Infection, Recombination, and Gene Loss’, The Journal of Infectious Diseases, 220: 781–91. 31050742 10.1093/infdis/jiz208 PMC6667795

[veaa099-B36] Suárez N. M. et al (2019b) ‘ Multiple-Strain Infections of Human Cytomegalovirus with High Genomic Diversity Are Common in Breast Milk from Human Immunodeficiency Virus-Infected Women in Zambia’, The Journal of Infectious Diseases, 220: 792–801. 31050737 10.1093/infdis/jiz209 PMC6667993

[veaa099-B37] Suárez N. M. et al (2020) ‘ Whole-Genome Approach to Assessing Human Cytomegalovirus Dynamics in Transplant Patients Undergoing Antiviral Therapy’, Frontiers in Cellular and Infection Microbiology, 10: 1–12. 32612959 10.3389/fcimb.2020.00267 PMC7308726

[veaa099-B38] Wilm A. et al (2012) ‘ LoFreq: A Sequence-Quality Aware, Ultra-Sensitive Variant Caller for Uncovering Cell-Population Heterogeneity from High-Throughput Sequencing Datasets’, Nucleic Acids Research, 40: 11189–201. 23066108 10.1093/nar/gks918 PMC3526318

[veaa099-B39] Xu H. et al (2012) ‘ FastUniq: A Fast De Novo Duplicates Removal Tool for Paired Short Reads’, PLoS One, 7: e52249. 23284954 10.1371/journal.pone.0052249 PMC3527383

[veaa099-B40] Yue J. X., Liti G., Hancock J. (2019) ‘ SimuG: A General-Purpose Genome Simulator’, Bioinformatics, 35: 4442–4. 31116378 10.1093/bioinformatics/btz424 PMC6821417

[veaa099-B41] Zuhair M. et al (2019) ‘ Estimation of the Worldwide Seroprevalence of Cytomegalovirus: A Systematic Review and Meta-Analysis’, Reviews in Medical Virology, 29: e2034–6. 30706584 10.1002/rmv.2034

