# Supplementary material for: GRACy: A tool for analysing human cytomegalovirus sequence data
Source: Virus Evol. 2020 Dec 30;7(1):veaa099. doi: 10.1093/ve/veaa099 (PMC7816668; doi:10.1093/ve/veaa099)
Supplement: veaa099_Supplementary_Data [file veaa099_supplementary_data.zip › Table S1.docx]

**Table S1**. Statistics of primary simulated datasets aligned to Merlin or MerlinVar.

| **Dataset name** | **Reads (no.)** | **Coverage depth mean ± SD^a^ (reads/nt)** | **Coverage breadth (nt)**b |
| --- | --- | --- | --- |
| merlinEC | 3,200,000 | 2,031.6 ± 77.2 | 235,646 |
| merlinUC | 5,758,626 | 3,656.0 ± 1,549.5 | 235,646 |
| merlinVarEC | 3,200,000 | 2,031.6 ± 53.1 | 235,646 |
| merlinVarUC | 5,570,328 | 3,536.4 ± 1605.7 | 235,646 |

^a^Abbreviation: SD, standard deviation.

^b^No. of nt in Merlin or MerlinVar aligned to ≥1 read; maximum = 235,646 nt.
