# Supplementary material for: GRACy: A tool for analysing human cytomegalovirus sequence data
Source: Virus Evol. 2020 Dec 30;7(1):veaa099. doi: 10.1093/ve/veaa099 (PMC7816668; doi:10.1093/ve/veaa099)
Supplement: veaa099_Supplementary_Data [file veaa099_supplementary_data.zip › Table S2.docx]

**Table S2**. Statistics of datasets subsampled from the primary simulated datasets and aligned to Merlin or MerlinVar.

| **Dataset name** | **Reads (no.)** | **Coverage depth mean ± SD^a^ (reads/nt)** | **Coverage breadth (nt)^b^** |
| --- | --- | --- | --- |
| merlin_EC_^20k^ | 20,000 | 12.7 ± 3.6 | 235,615 |
| merlin_EC_^40k^ | 40,000 | 25.4 ± 5.1 | 235,628 |
| merlin_EC_^60k^ | 60,000 | 38.1 ± 6.3 | 235,628 |
| merlin_EC_^80k^ | 80,000 | 50.8 ± 7.3 | 235,635 |
| merlin_EC_^100k^ | 100,000 | 63.5 ± 8.3 | 235,635 |
| merlin_EC_^150k^ | 150,000 | 95.2 ± 10.3 | 235,646 |
| merlin_EC_^200k^ | 200,000 | 127.0 ± 12.2 | 235,646 |
| merlin_EC_^400k^ | 400,000 | 254.0 ± 17.7 | 235,646 |
| merlin_EC_^800k^ | 800,000 | 507.9 ± 27.4 | 235,646 |
| merlin_EC_^1600k^ | 1,600,000 | 1015.8 ± 45.1 | 235,646 |
| merlin_EC_^2400k^ | 2,400,000 | 1523.7 ± 61.4 | 235,646 |
| merlin_EC_^3200k^ | 3,200,000 | 2031.6 ± 77.2 | 235,646 |
| merlin_UC_^20k^ | 20,000 | 12.8 ± 6.4 | 232,894 |
| merlin_UC_^40k^ | 40,000 | 25.5 ± 11.8 | 235,198 |
| merlin_UC_^60k^ | 60,000 | 38.2 ± 17.2 | 235,371 |
| merlin_UC_^80k^ | 80,000 | 50.9 ± 22.5 | 235,583 |
| merlin_UC_^100k^ | 100,000 | 63.6 ± 27.9 | 235,599 |
| merlin_UC_^150k^ | 150,000 | 95.4 ± 41.3 | 235,621 |
| merlin_UC_^200k^ | 200,000 | 127.1 ± 54.4 | 235,630 |
| merlin_UC_^400k^ | 400,000 | 254.0 ± 108.1 | 235,640 |
| merlin_UC_^800k^ | 800,000 | 507.9 ± 215.6 | 235,646 |
| merlin_UC_^1600k^ | 1,600,000 | 1015.8 ± 431.0 | 235,646 |
| merlin_UC_^2400k^ | 2,400,000 | 1523.7 ± 645.4 | 235,646 |
| merlin_UC_^3200k^ | 3,200,000 | 2031.6 ± 860.7 | 235,646 |
| merlinVar_EC_^20k^ | 20,000 | 12.7 ± 3.6 | 235,611 |
| merlinVar_EC_^40k^ | 40,000 | 25.4 ± 5.1 | 235,628 |
| merlinVar_EC_^60k^ | 60,000 | 38.1 ± 6.3 | 235,641 |
| merlinVar_EC_^80k^ | 80,000 | 50.8 ± 7.3 | 235,641 |
| merlinVar_EC_^100k^ | 100,000 | 63.5 ± 8.1 | 235,641 |
| merlinVar_EC_^150k^ | 150,000 | 95.2 ± 10.1 | 235,641 |
| merlinVar_EC_^200k^ | 200,000 | 127.0 ± 12.0 | 235,641 |
| merlinVar_EC_^400k^ | 400,000 | 254.0 ± 18.1 | 235,644 |
| merlinVar_EC_^800k^ | 800,000 | 507.9 ± 28.1 | 235,645 |
| merlinVar_EC_^1600k^ | 1,600,000 | 1,015.8 ± 47.8 | 235,646 |
| merlinVar_EC_^2400k^ | 2,400,000 | 1,523.7 ± 65.8 | 235,646 |
| merlinVar_EC_^3200k^ | 3,200,000 | 1,993.5 ± 81.9 | 235,646 |
| merlinVar_UC_^20k^ | 20,000 | 12.9 ± 6.7 | 232,740 |
| merlinVar_UC_^40k^ | 40,000 | 25.5 ± 12.4 | 235,010 |
| merlinVar_UC_^60k^ | 60,000 | 38.1 ± 18.1 | 235,479 |
| merlinVar_UC_^80k^ | 80,000 | 50.8 ± 23.9 | 235,592 |
| merlinVar_UC_^100k^ | 100,000 | 63.5 ± 29.5 | 235,602 |
| merlinVar_UC_^150k^ | 150,000 | 95.2 ± 44.0 | 235,614 |
| merlinVar_UC_^200k^ | 200,000 | 127.0 ± 58.6 | 235,635 |
| merlinVar_UC_^400k^ | 400,000 | 254.0 ± 116.2 | 235,644 |
| merlinVar_UC_^800k^ | 800,000 | 507.9 ± 231.6 | 235,646 |
| merlinVar_UC_^1600k^ | 1,600,000 | 1,015.8 ± 462.2 | 235,646 |
| merlinVar_UC_^2400k^ | 2,400,000 | 1,523.7 ± 693.1 | 235,646 |
| merlinVar_UC_^3200k^ | 3,200,000 | 2,031.6 ± 923.0 | 235,646 |

^a^Abbreviation: SD, standard deviation.

^b^No. of nt in Merlin or MerlinVar aligned to ≥1 read; maximum = 235,646 nt.
