# Supplementary material for: GRACy: A tool for analysing human cytomegalovirus sequence data
Source: Virus Evol. 2020 Dec 30;7(1):veaa099. doi: 10.1093/ve/veaa099 (PMC7816668; doi:10.1093/ve/veaa099)
Supplement: veaa099_Supplementary_Data [file veaa099_supplementary_data.zip › Table S3.docx]

**Table S3**. Statistics of datasets simulating two mixed strains aligned to Merlin or MerlinVar.

| **Dataset name** | **Reads (no.)^a^** | **Minor strain (%)** | **Coverage depth mean (reads/nt)** | **Nucleotides with coverage depth <10 reads/nt (nt)** | **Coverage breadth (nt)^b^** |
| --- | --- | --- | --- | --- | --- |
| mixedStrains_EC_^28k^ | 28,000 | 0.9 | 17.8 | 3792 | 235,628 |
| mixedStrains_EC_^56k^ | 56,000 | 1.7 | 35.5 | 120 | 235,641 |
| mixedStrains_EC_^112k^ | 112,000 | 3.4 | 71.1 | 54 | 235,646 |
| mixedStrains_EC_^224k^ | 224,000 | 6.5 | 142.2 | 39 | 235,646 |
| mixedStrains_EC_^280k^ | 280,000 | 8.0 | 177.7 | 31 | 235,646 |
| mixedStrains_EC_^560k^ | 560,000 | 14.9 | 355.5 | 9 | 235,646 |
| mixedStrains_EC_^1120k^ | 1,120,000 | 25.9 | 710.9 | 2 | 235,646 |
| mixedStrains_UC_^28k^ | 28,000 | 0.9 | 17.9 | 47,357 | 234,465 |
| mixedStrains_UC_^56k^ | 56,000 | 1.7 | 35.6 | 15,532 | 235,361 |
| mixedStrains_UC_^112k^ | 112,000 | 3.4 | 71.2 | 4,463 | 235,593 |
| mixedStrains_UC_^224k^ | 224,000 | 6.5 | 142.2 | 1,144 | 235,642 |
| mixedStrains_UC_^280k^ | 280,000 | 8.0 | 177.8 | 599 | 235,644 |
| mixedStrains_UC_^560k^ | 560,000 | 14.9 | 355.5 | 126 | 235,645 |
| mixedStrains_UC_^1120k^ | 1,120,000 | 25.9 | 711.0 | 29 | 235,646 |

^a^No. of merlinVar_EC_ or merlinVar_UC_ reads added to merlin_EC_^3200k^ or merlin_UC_^3200k^, respectively.

^b^No. of nt in Merlin or MerlinVar aligned to ≥1 read; maximum = 235,646 nt.
