# Supplementary material for: GRACy: A tool for analysing human cytomegalovirus sequence data
Source: Virus Evol. 2020 Dec 30;7(1):veaa099. doi: 10.1093/ve/veaa099 (PMC7816668; doi:10.1093/ve/veaa099)
Supplement: veaa099_Supplementary_Data [file veaa099_supplementary_data.zip › Table S4.docx]

**Table S4:** Features of experimental datasets.

| **Dataset name** | **Genome accession no. (GenBank)** | **Dataset accession no. (ENA)** | **Reads (no.)** | **Viral load (IU/ml)** | **Genome copies for library (no.)** |
| --- | --- | --- | --- | --- | --- |
| JER4755^a^ | KR534209.1 | ERR3014430 | 3,330,196 | 2,101,650 | 21,017 |
| JER8451^a^ | KR534197.1 | ERR3013936 | 879,526 | Unknown | Unknown |
| PAV25^a^ | KJ361969.1 | ERR3014175 | 4,066,506 | 1,760,000 | 8,000 |
| PAV6^a^ | KJ361962.1 | ERR3013919 | 4,877,134 | 160,000 | 88,000 |
| Lon2_T1^b^ | KT726948.2 | ERR1279035 | 1,863,532 | 333,831 | Unspecified |
| Lon2_T2^b^ | KT726948.2 | ERR1279043 | 1,888,564 | 5,114,408 | Unspecified |
| Lon2_T3^b^ | KT726948.2 | ERR1279044 | 2,571,696 | 3,528,590 | Unspecified |

^a^Reported in (Suarez et al. 2019).

^b^Reported in (Houldcroft et al., 2016a): these datasets correspond to samples from patient I.
