# Supplementary material for: GRACy: A tool for analysing human cytomegalovirus sequence data
Source: Virus Evol. 2020 Dec 30;7(1):veaa099. doi: 10.1093/ve/veaa099 (PMC7816668; doi:10.1093/ve/veaa099)
Supplement: veaa099_Supplementary_Data [file veaa099_supplementary_data.zip › Supplemental File 1.docx]

**Software implementation**

GRACy opens a window accessing six modules that perform the following tasks: read filtering, genotyping, genome assembly, genome annotation, variant analysis and database submission. This document provides selected additional information on their operation.

**1. Read filtering**

This module can greatly improve the performance of downstream steps. It involves removing reads that did not originate from the organism under study, removing the sequences of adapters used during sequencing library construction, and removing clonal reads (deduplication) generated from the same genome fragments.

The step in which human reads are removed relies on a an alignment to the human reference genome which initially generates a sam file. Conversion to bam format is achieved using this command:

*samtools view -bS -h alignment.sam > alignment.bam*

Unmapped (non-host) reads are then extracted in fastq format using this command:

*bam2fastq --no-aligned --force --strict -o output#.fastq alignment.bam*

The reads can be filtered for default or user-selected adapters and for quality using Trim Galore. Deduplication can be performed using FastUniq with default parameters. Alignment of the reads to the Merlin genome can be performed in order to calculate the average depth and breadth of coverage. This is achieved using this pipeline:

1. Align the reads to the Merlin genome using Bowtie 2.
2. Convert the alignment from sam to bam format using the command specified above.
3. Sort the bam file using this command:

*samtools sort -o alignment_sorted.bam alignment.bam*

1. Calculate the average depth and breadth of coverage using these commands:

*samtools depth -d 10000000 alignment_sorted.bam | awk '{sum+=$3} END { print sum/NR}' >coverage.txt*

*samtools depth -d 10000000 alignment_sorted.bam | wc -l >breadth.txt*

Coverage plots are produced by internal Python scripts based on the matplotlib library.

**2. Genotyping**

This module analyses the genotypes of a selection of hypervariable HCMV genes, and is especially useful for characterising samples containing multiple HCMV strains. The process starts by estimating coverage depth across the genome. Initially, the reads are aligned to the Merlin genome, and the coverage characteristics are calculated as described above. As the presence of hypervariable genes is likely to result in portions of the genome not being covered by reads, the average coverage values are calculated by considering only the positions that are covered by ≥1 read. Reads are then deduplicated using FastUniq, and all possible kmers (k = 17) within them are counted using this command:

*jellyfish count -m 17 -C fastqFile_1.fastq fastqFile_2.fastq*

This kmer size was chosen because it provides an optimal compromise between sensitivity and accuracy. Thus, higher values improve accuracy but result in the absence of specific kmers for some genotypes, and lower values improve sensitivity but increase the susceptibility of kmers to sequence error artefacts.

A dictionary is created associating each kmer with the reads in which it is present. A dataset of genotype-specific kmers in a set of hypervariable genes is available within the software and is searched for all kmers that are specific to a particular gene and genotype using this command:

*jellyfish query*

The reads containing relevant kmers are combined in a nonredundant list. If the number of reads exceeds a user-defined proportion of the estimated coverage depth, the genotype is reported in the output file. For each hypervariable gene, any genotype is reported as the ratio between the total number of reads calling that genotype and the total number of reads calling any genotype for that gene.

**3. Genome assembly**

This module assembles an HCMV genome *de novo* from the reads. It consists of six steps that can be implemented individually using a configuration file (see the manual within the GRACy distribution).

**3.1. Read filtering**

This step further improves dataset quality using PRINSEQ with the parameters provided in the configuration file.

**3.2. *De novo* assembly**

This step assembles the reads into contigs. Quality-filtered reads in a paired-end dataset are concatenated using this command:

*interleave-reads.py*

and normalized using this command:

*normalize-by-median.py -k 17 -C 200 -M AvailableMemory -p*

with the AvailableMemory parameter provided by the user. Both of these scripts are included in the khmer package.

The normalized reads are subsampled from the full dataset in proportions of 0.2-1. Each subsampled dataset is assembled using SPAdes with the parameters *--cov-cutoff auto --careful -k 53,63,73,83*, each time producing a different contig set. The contig set featuring the highest N50 value is used to perform subsequent steps. The N50 value is associated with the length of the assembled contigs but not to their accuracy, which is instead accounted for in the following steps.

**3.3. Scaffolding**

This step scaffolds the contigs generated in the previous step on the basis of relative position and orientation. This is achieved using Ragout with the Merlin genome as reference. At this stage, a single sequence is produced that is hereafter referred to as the preliminary assembly. Since the presence of inverted repeats in the HCMV genome frequently prompts misassembly, GRACy attempts to reconstruct a 10,000 nt region incorporating the whole of *b’a’c’* (approximately 190,000-200,000 nt in the Merlin genome), which is hereafter referred to as the central repeat region, using this pipeline:

1. The reads are aligned to the central repeat region in the Merlin genome.
2. The alignment file is converted to sam format and sorted as described above.
3. Read pairs for which at least one end is aligned are extracted using bam2fastq.
4. The extracted reads are assembled *de novo* using SPAdes with the parameters *--cov-cutoff auto --careful -k 51,61,71.*
5. The resulting contigs are scaffolded against the Merlin sequence (190,000-200,000 nt) using Scaffold_builder.
6. The scaffolded sequence is aligned with the Merlin sequence (190,000-200,000 nt) using LASTZ v. 1.04 (Harris, R.S. (2007). Improved pairwise alignment of genomic DNA. Ph.D. Thesis, The Pennsylvania State University), and only the portion included in the alignment is retained. This step produces the central repeat region.
7. If ambiguous nucleotides are present, the sequence is improved using Gapfiller.

The reconstructed central repeat region is generally more accurate than that obtained using Ragout, and is used to replace the corresponding region in the preliminary assembly. To resolve gaps due to low coverage depth, the flanking regions 200 nt on each side of a gap are located within a large collection of HCMV genomes using blastn. If close similarity is found for both flanking regions to the same genome (e.g. each alignment covers at least 100 nucleotides with e < 0.0001), the reads are aligned with this region, substitutions and indels are corrected, and the consensus is incorporated into the preliminary assembly, using Ns for any region lacking coverage. Attempts are made to fill remaining gaps using Gapfiller.

**3.4. First consensus call**

This step resolves remaining minor errors involving substitutions or indels in the preliminary assembly. The reads are aligned to this sequence using Bowtie 2 with default parameters. Duplicate reads in the alignment file are marked (i.e. a new alignment file is generated in which a flag is added to duplicate reads) using these commands:

*picard AddOrReplaceReadGroups*

*picard MarkDuplicates*

Briefly, a read pair derived clonally from the same DNA fragment as a previous read pair is identified as sharing the same mapping positions, and one of them is marked as a duplicate. All read pairs marked as duplicates are removed using this command:

*samtools view -F 1024*

and the resulting bam file is used to produce a pileup file using this command:

*samtools mpileup*

Single nucleotide polymorphisms (SNPs) and insertions or deletions (indels) are called from this file using this command:

*varscan mpileup2csn*

with the parameters *--variants --output-vcf --min-var-freq 0.5*. This generates a vcf variants file that is zipped using bgzip and indexed using tabix, and used to amend the preliminary assembly for any SNP or indel detected at a frequency of >50%, using this command:

*bcftools consensus*

As SNPs and indels may not be called in repeat regions due to ambiguous mapping, the pipeline performs polymorphism detection on these regions individually.

**3.5. Assembly refinement**

This step corrects remaining misassembly errors in the preliminary assembly with SNPs and indels amended. The reads are aligned to this sequence as described above, and a pileup file reporting coverage information at each position is obtained using this command:

*samtools mpileup*

Regions with a coverage depth of <3 reads/nt are considered to represent potential misassembly errors and are removed and replaced by gaps. Attempts to fill these gaps are performed using an overlap-layout-consensus approach. Briefly, the regions flanking the gaps (1500 nt each) are extracted, thus producing two sequences referred to as the 5’-gapFlanking and 3’gapFlanking sequences. The original reads are aligned to the 5’-gapFlanking sequence using Bowtie 2 with parameters *--local --very-sensitive-local*. Reads mapping at the 3’ end of the 5’-gapFlanking sequence that fulfil the following conditions are then identified: (i) they align without gaps, (ii) they extend beyond the 3’ end of the 5’gapFlanking sequence by ≥40 nt, and (iii) their mates maps for their entire length within the 5’-gapFlanking sequence. These soft-clipped reads are clustered using cd-hit-est, and those belonging to the most abundant cluster are reported in fasta format (this step is important for avoiding the extension of the 5-gapFlanking sequence using data from a minor HCMV strain represented in the dataset). These reads are assembled using cap3, and the resulting contig is used to extend the original 5’-gapFlanking sequence. This process is repeated until the 3’gapFlanking sequence is reached, as indicated by a valid blast alignment of ≥30nt between the extending sequence and the 3’gapFlanking sequence. Finally, the reconstructed sequence is used to replace the original gap.

**3.6. Second consensus call**

This step resolves substitutions or indels introduced in the previous step by repeating the first consensus call step and thus generating the final assembly.

**4. Genome annotation**

This module generates an annotation of a HCMV genome by searching for the 170 CDSs annotated in the Merlin genome and returning the coordinates. For each CDS, a fasta file is provided within the module reporting all the sequences available in the Uniprot database (1 December 2019). This file is used as a query in a tblastn search of the finished genome in order to find the most closely matching protein candidate. The sequence of this protein is then used to search for the corresponding CDS using this command:

*exonerate --model protein2genome proteinFastaFile.fasta targetGenomeFastaFile.fasta --showtargetgff -s 0 -n 1 --forcegtag --minintron 35 --maxintron 10000*

The sequence identified is checked for the presence of a valid start codon and stop codon, and further rounds of refinement are carried out if necessary.

**5. Variant analysis**

This module uses PRINSEQ and LoFreq thresholds derived from extensive testing of several simulated and real datasets. An *ad hoc* script uses the SNP data (in vcf format), together with a reference genome, the CDSs (in fasta format) and an annotation file (in gff format) to map variants within CDSs and report their effects on coding potential.

**6. Database submission**

This module supports submission of datasets to the European Nucleotide Archive (ENA). Each dataset (termed Run) can be submitted within an ENA sample that, in turn, can be submitted as a part of an ENA Project or Study. The user may create a new Study or a new Sample (or both) for a dataset. Project and Sample information are provided in the form of tab-delimited text files that are used to produce submission files in xml format. An example of a Project file (project.xml) is as follows:

*<PROJECT_SET>*

*<PROJECT alias="Zambian_milk_HCMV">*

*<NAME>Zambian_milk_HCMV</NAME>*

*<TITLE>HTS data for HCMV</TITLE>*

*<DESCRIPTION>HTS data for HCMV</DESCRIPTION>*

*<SUBMISSION_PROJECT>*

*<SEQUENCING_PROJECT></SEQUENCING_PROJECT>*

*</SUBMISSION_PROJECT>*

*<PROJECT_LINKS>*

*<PROJECT_LINK>*

*<XREF_LINK>*

*<DB></DB>*

*<ID></ID>*

*</XREF_LINK>*

*</PROJECT_LINK>*

*</PROJECT_LINKS>*

*</PROJECT>*

*</PROJECT_SET>*

The Project is created in the ENA database using this command:

*curl -u Username:password -F "SUBMISSION=@submission.xml" -F "PROJECT=@project.xml" "https://www.ebi.ac.uk/ena/submit/drop-box/submit*/"

An example of a Sample file (sample.xml) is as follows:

*<?xml version="1.0" encoding="UTF-8"?>*

*<SAMPLE_SET>*

*<SAMPLE alias="SYD-2_T10" center_name="UNIVERSITY OF GLASGOW">*

*<TITLE>SYD-2 longitudinal sample</TITLE>*

*<SAMPLE_NAME>*

*<TAXON_ID>10359</TAXON_ID>*

*<SCIENTIFIC_NAME>Human betaherpesvirus 5</SCIENTIFIC_NAME>*

*<COMMON_NAME></COMMON_NAME>*

*</SAMPLE_NAME>*

*<SAMPLE_ATTRIBUTES>*

*<SAMPLE_ATTRIBUTE>*

*<TAG>geographic location (country and/or sea)</TAG>*

*<VALUE>Australia</VALUE>*

*</SAMPLE_ATTRIBUTE>*

*<SAMPLE_ATTRIBUTE>*

*<TAG>host common name</TAG>*

*<VALUE>human</VALUE>*

*</SAMPLE_ATTRIBUTE>*

*<SAMPLE_ATTRIBUTE>*

*<TAG>host health state</TAG>*

*<VALUE>transplant patient</VALUE>*

*</SAMPLE_ATTRIBUTE>*

*<SAMPLE_ATTRIBUTE>*

*<TAG>isolation source host associated</TAG>*

*<VALUE>plasma</VALUE>*

*</SAMPLE_ATTRIBUTE>*

*<SAMPLE_ATTRIBUTE>*

*<TAG>host sex</TAG>*

*<VALUE>Unspecified</VALUE>*

*</SAMPLE_ATTRIBUTE>*

*<SAMPLE_ATTRIBUTE>*

*<TAG>host scientific name</TAG>*

*<VALUE>homo sapiens</VALUE>*

*</SAMPLE_ATTRIBUTE>*

*<SAMPLE_ATTRIBUTE>*

*<TAG>collector name</TAG>*

*<VALUE>Unspecified</VALUE>*

*</SAMPLE_ATTRIBUTE>*

*<SAMPLE_ATTRIBUTE>*

*<TAG>collecting institution</TAG>*

*<VALUE>WESTMEAD HOSPITAL</VALUE>*

*</SAMPLE_ATTRIBUTE>*

*<SAMPLE_ATTRIBUTE>*

*<TAG>isolate</TAG>*

*<VALUE>SYD-2</VALUE>*

*</SAMPLE_ATTRIBUTE>*

*</SAMPLE_ATTRIBUTES>*

*</SAMPLE>*

*</SAMPLE_SET>*

The Sample is created in the ENA database using this command:

*curl -u Username:password -F "SUBMISSION=@submission.xml" -F "Sample=@sample.xml" "https://www.ebi.ac.uk/ena/submit/drop-box/submit/"*

After the datasets have been copied to the ENA web space (see https://ena-docs.readthedocs.io/en/latest/submit/fileprep/upload.html), the submission is finalized by creating a manifest file for each Sample that contains information provided by the user. An example of a manifest file for a Sample is as follows:

INSTRUMENT Illumina MiSeq

INSERT_SIZE 500

LIBRARY_SOURCE GENOMIC

LIBRARY_SELECTION hybrid selection

STUDY PRJEB36759

SAMPLE ERS4307365

NAME SYD-2_T10

LIBRARY_STRATEGY WGS

FASTQ SYD-2_T10_purged_1.fastq.gz

FASTQ SYD-2_T10_purged_2.fastq.gz

This file is submitted using webin-cli-2.2.0.jar, which is the official ENA tool for program-driven dataset submission. Any errors during Project, Sample or Run submission are reported to the user.
