# Supplementary figures and images for: GRACy: A tool for analysing human cytomegalovirus sequence data
Source: Virus Evol. 2020 Dec 30;7(1):veaa099. doi: 10.1093/ve/veaa099 (PMC7816668; doi:10.1093/ve/veaa099)

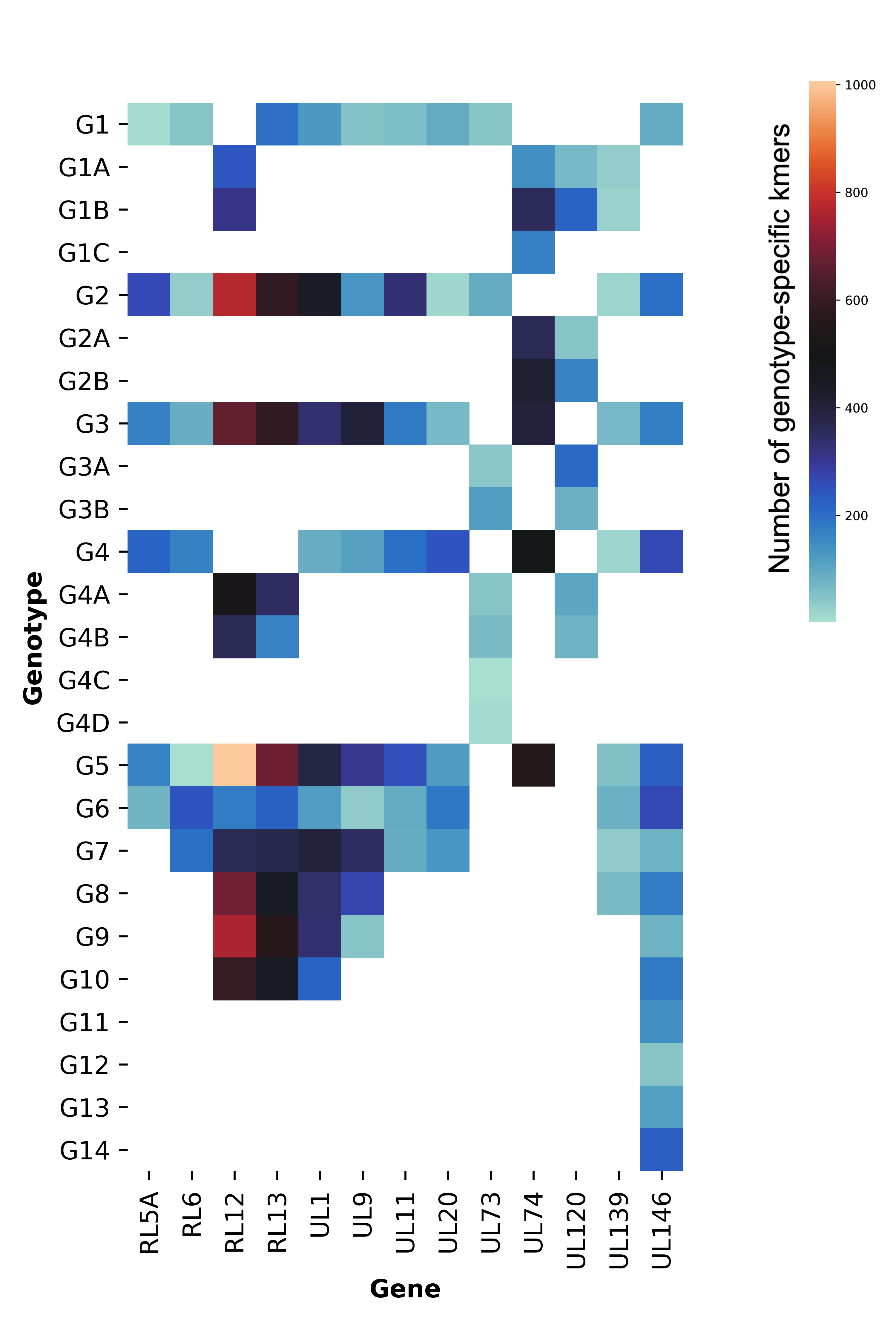

Supplement: veaa099_Supplementary_Data [file veaa099_supplementary_data.zip › Figure S1.jpg]

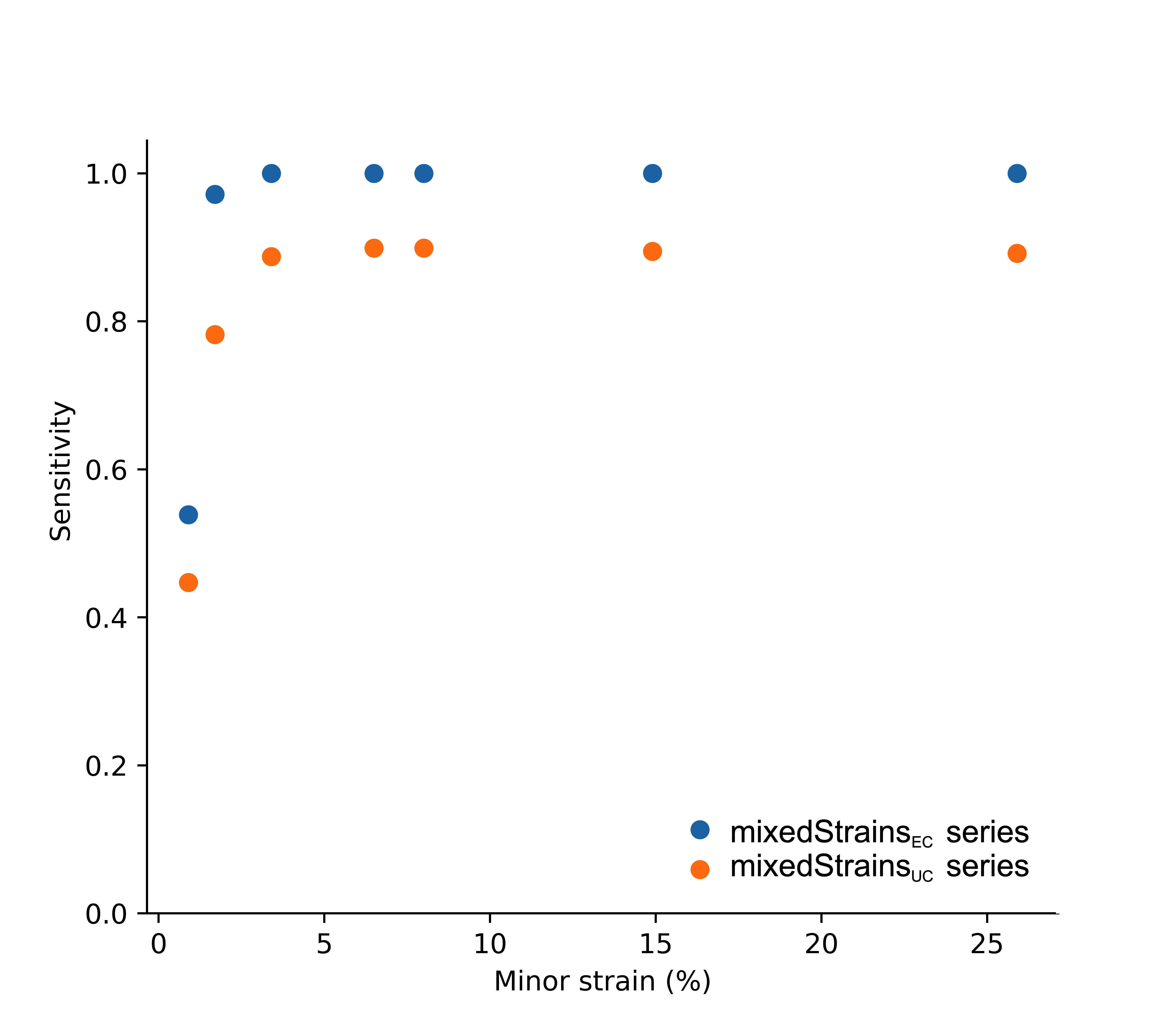

Supplement: veaa099_Supplementary_Data [file veaa099_supplementary_data.zip › Figure S2.png]

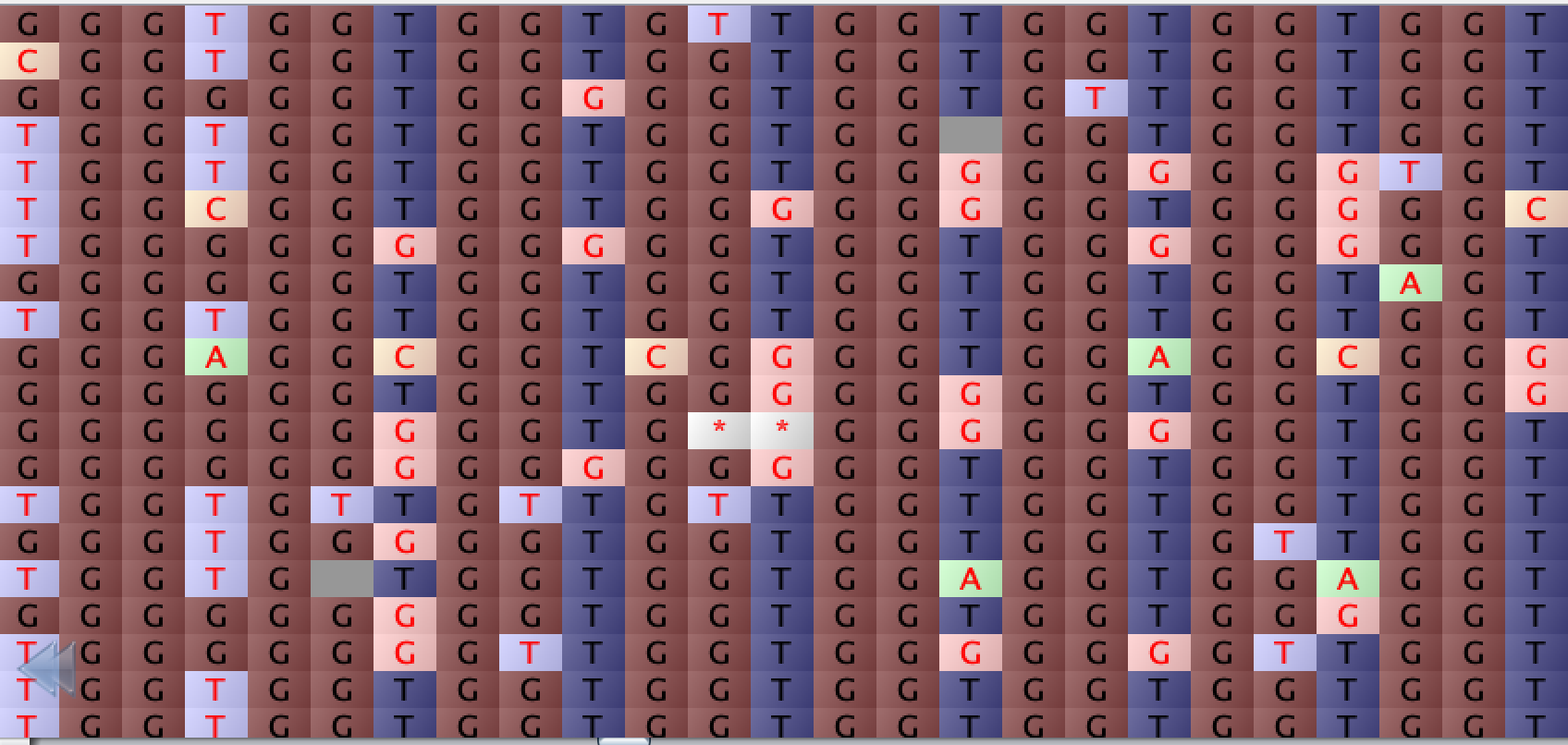

Supplement: veaa099_Supplementary_Data [file veaa099_supplementary_data.zip › Figure S3.png]
